# Integrated Care Intervention Supported by a Mobile Health Tool for Patients Using Noninvasive Ventilation at Home: Randomized Controlled Trial

**DOI:** 10.2196/16395

**Published:** 2020-04-13

**Authors:** Erik Baltaxe, Cristina Embid, Eva Aumatell, María Martínez, Anael Barberan-Garcia, John Kelly, John Eaglesham, Carmen Herranz, Eloisa Vargiu, Josep Maria Montserrat, Josep Roca, Isaac Cano

**Affiliations:** 1 Hospital Clínic de Barcelona Institut d’Investigacions Biomèdiques August Pi i Sunyer Universitat de Barcelona Barcelona Spain; 2 Center for Biomedical Research Network in Respiratory Diseases Madrid Spain; 3 Advanced Digital Innovation (UK) Ltd Salts Mill United Kingdom; 4 Eurecat Technological Center of Catalonia Barcelona Spain

**Keywords:** behavioral change, eHealth, noninvasive ventilation, mobile health, chronic diseases

## Abstract

**Background:**

Home-based noninvasive ventilation has proven cost-effective. But, adherence to therapy still constitutes a common clinical problem. We hypothesized that a behavioral intervention supported by a mobile health (mHealth) app could enhance patient self-efficacy. It is widely accepted that mHealth-supported services can enhance productive interactions among the stakeholders involved in home-based respiratory therapies.

**Objective:**

This study aimed to measure changes in self-efficacy in patients with chronic respiratory failure due to diverse etiologies during a 3-month follow-up period after the intervention. Ancillary objectives were assessment of usability and acceptability of the mobile app as well as its potential contribution to collaborative work among stakeholders.

**Methods:**

A single-blind, single-center, randomized controlled trial was conducted between February 2019 and June 2019 with 67 adult patients with chronic respiratory failure undergoing home-based noninvasive ventilation. In the intervention group, a psychologist delivered a face-to-face motivational intervention. Follow-up was supported by a mobile app that allowed patients to report the number of hours of daily noninvasive ventilation use and problems with the therapy. Advice was automatically delivered by the mobile app in case of a reported problem. The control group received usual care. The primary outcome was the change in the Self Efficacy in Sleep Apnea questionnaire score. Secondary outcomes included app usability, app acceptability, continuity of care, person-centered care, and ventilatory parameters.

**Results:**

Self-efficacy was not significantly different in the intervention group after the intervention (before: mean 3.4, SD 0.6; after: mean 3.4, SD 0.5, *P*=.51). No changes were observed in adherence to therapy nor quality of life. Overall, the mHealth tool had a good usability score (mean 78 points) and high acceptance rate (mean score of 7.5/10 on a Likert scale). It was considered user-friendly (mean score of 8.2/10 on a Likert scale) and easy to use without assistance (mean score of 8.5/10 on a Likert scale). Patients also scored the perception of continuity of care and person-centered care as high.

**Conclusions:**

The integrated care intervention supported by the mobile app did not improve patient self-management. However, the high acceptance of the mobile app might indicate potential for enhanced communication among stakeholders. The study identified key elements required for mHealth tools to provide effective support to collaborative work and personalized care.

**Trial Registration:**

ClinicalTrials.gov NCT03932175; https://clinicaltrials.gov/ct2/show/NCT03932175

## Introduction

In the 1950s, the polio epidemic demonstrated the safety and efficacy of noninvasive ventilation (NIV) to decrease mortality [1]. Since then, the use of this therapeutic approach at home has reduced hospital admissions, has favorably impacted health-related quality of life, improved sleep quality, and reduced mortality in patients with chronic respiratory failure due to diverse etiologies [2-8]. These results have driven a steady increase in the prevalence of patients using home-based NIV in Europe, ranging from 4.5 to 20 per 100,000 adults [9-11].

Despite its proven cost-effectiveness [12], patient adherence to home-based NIV could still improve, which should further enhance health care–related efficiencies of the intervention [13]. Monitoring and optimization of physiological settings have enhanced adherence by improving the timely detection of problems such as mask leaks and patient-ventilator asynchronies [14]. Nevertheless, improvement in the behavioral aspects such as patient motivation and empowerment for self-management are important factors to consider when addressing adherence to respiratory therapies.

The current study sought to explore the transfer of previous positive experiences with behavioral interventions in other fields (ie, physical activity) [15-20] to home-based NIV. Specifically, we addressed the concept of self-efficacy, defined as the individual’s perceived capability to perform a particular behavior [21]. Self-efficacy expectations can be affected by enablers or barriers such as the perception of physical function or the capacity for self-management. Therefore, a person who does not believe in her or his capacity to perform the desired action will fail to adopt, initiate, and maintain it. Self-efficacy is therefore seen as the most influential motivational factor and the strongest predictor of behavioral intentions [21].

We propose the use of a behavioral mobile health (mHealth) intervention, which can be framed by Bandura’s model [22], to support changes in self-efficacy. This model is based on the concepts of health risk perceptions, health outcome expectancies, and the patients’ confidence to engage in certain behaviors. The model has been widely applied in studies of the adoption, initiation, and maintenance of health-promoting behaviors [23].

In addition to self-efficacy as a way to influence behavioral change, previous reports by Hernandez et al [24] and Cano et al [25] identified two common hinderances for the effective implementation of complex respiratory therapies (ie, long-term oxygen therapy, continuous positive airway pressure therapy, home NIV, and home-based nebulizer therapy). First, interaction and communication, which could greatly benefit from digital tools supporting collaborative work, are needed among several stakeholders, namely health professionals at different health care tiers (eg, primary care, specialized care), patients and carers, companies undertaking equipment maintenance, and others. Second, improvement in therapeutic adherence is needed, which could be achieved by empowering patients to perform self-management.

Within this context, information and communication technologies (ICT) have been identified as promising tools to enhance the coordination between stakeholders and contribute to improved health outcomes [26,27]. Nonetheless, the implementation remains immature [28] due to a lack of evidence in a real-world context for the capacity of ICT to sustain behavioral changes, including self-efficacy, in patients with chronic, complex conditions. It is widely accepted that, despite current limitations, patients with chronic, complex conditions are an ideal population for which care coordination, patient and medical staff satisfaction, and patient empowerment are of the utmost importance to produce health benefits.

The principal objective of this study was to explore the capacity of a behavioral mHealth intervention to increase patient empowerment for self-management and adherence to therapy. The secondary aim was to learn, based on the experience of professionals and patients, how the mHealth tool should evolve to support collaborative work.

## Methods

### Study Design and Participants

A single-blind, single-center, randomized controlled trial with two parallel arms (1:1 ratio) was conducted. Patients were randomized to a control group or an intervention arm, which consisted of the behavioral mHealth intervention in addition to usual care. Inclusion criteria were as follows: all adult patients with hypercapnic ventilatory failure due to chest wall, neuromuscular, lung parenchyma, or airway disease already receiving treatment with NIV irrespective of treatment duration and in possession of a mobile phone or tablet that could support the use of the mHealth app (MyPathway). MyPathway [29] is a secure, digital communication channel connecting patients to clinicians and services. It is an app-based tool for both patients and clinicians to use on phones or tablets. See Multimedia Appendix 1 for more details. Patients with severe psychiatric or neurological diseases were excluded, as well as patients hospitalized at the time of assessment.

### Intervention

In addition to usual care, the behavioral mHealth intervention included a face-to-face motivational interview by a psychologist (EA) to assess the patient’s adherence profile and lifestyle habits, with a follow-up through the MyPathway app. In contrast, the control group received only usual care, which consisted of manual discharge and review of the NIV machine data by the treating pulmonologist and respiratory nurse. Respiratory parameters were changed, if needed, according to clinical data (anamnesis and physical examination) in addition to NIV data.

At the time of enrollment, semi-structured motivational interviews were conducted individually. Participants were asked about their treatment adaptation experience, lifestyle (physical activity and food habits), and use of ICT. In each session, field notes were taken anonymously, and no recordings were made. The intervention consisted of a 10-50–minute face-to-face session at the hospital or participants' home that followed the principles of a collaborative and evocative motivational interview, favoring the participant's autonomy. The techniques used were open questions, active listening, empathy, returning reflected thoughts, exploring a change in goals, summarizing, and giving feedback. Also, during the enrollment visit, patients were given verbal and written explanation on how to use the app. Free access was granted after receiving an invitation via the hospital health information system (SAP), which prompted the participant to register using an email address as the username. The app could also be downloaded to the carers’ phone in case the patient did not have a smartphone.

During the follow-up, the MyPathway app was used by study participants for bidirectional interaction with the research team. It consisted of positive feedback or reinforcement messages in response to the number of hours of NIV use reported by the patient daily. Also, general advice on specific NIV clinical problems was automatically provided by the app according to the patients’ weekly input. Additional educational material on physical activity, diet, and sleep hygiene could be accessed at any time via a dedicated link. A web-based clinical portal enabled the research team to monitor the patient-reported NIV hours of use and clinical problems. As indicated, a dedicated nurse with clinical and technical knowledge (one of the authors, MM) took the role of case manager to support collaborative work. She used the web-based portal to identify adherence problems and contacted participants via telephone or at home (for those with severe mobility problems) to enquire about and solve potential clinical or technical problems.

### Procedures and Study Outcomes

The primary outcome was a change in self-efficacy, as measured using the Self Efficacy in Sleep Apnea (SEMSA) questionnaire. The SEMSA is a US-designed self-report questionnaire comprised of 26 items that are rated from 1 to 4 on a 4-point Likert scale [30]. The arithmetic mean of the Likert rating for each participant is computed for the overall SEMSA score and each of the 3 factors. The total score ranges from 1 to 4. Higher scores indicate greater risk perception, higher benefit expectancy with treatment, and greater perceived self-efficacy [30].

Secondary outcomes included usability of the ICT tool, as measured using the System Usability Scale [31]; patient satisfaction, as measured using the Net Promoter Score [32] in addition to 3 custom general satisfaction questions measured on a Likert scale; continuity of care, as measured using the Nijmegen Continuity Questionnaire [33]; and the Person-Centred Coordinated Care Experience Questionnaire as described by Leijten et al [34]. Moreover, ventilator-specific data such as the mean hours of daily use, unintentional leaks (L/s), minute ventilation (L/min), tidal volume (mL), and backup rate (breaths/min) were downloaded directly from the NIV machine.

Tertiary outcomes included mortality; health-related quality of life, as measured using the EuroQol 5D questionnaire [35,36]; and sleepiness, as measured using the Epworth Sleepiness Score.

The impact of the motivational mHealth tool recommendations on diet and exercise was indirectly measured by body weight changes.

All assessments were completed at baseline and the final visit scheduled 3 months later. The follow-up was conducted in the outpatient clinic for the control group and remotely by the nurse case manager (MM) using the MyPathway app and its clinical portal for the intervention group. When deemed necessary, the nurse case manager visited the patient at home, or a visit was scheduled in the outpatient clinics. There was no active follow-up for the control group.

### Randomization and Masking

All eligible patients were contacted by telephone to briefly explain the study and invite them to participate. Those showing interest were invited to the hospital outpatient clinics. Study investigators (EB, EA, and MM) explained the study face-to-face, and, in case of acceptance, signed consent was obtained. Afterward, the patient was randomized. Before patient enrollment, the randomization scheme was generated using the website randomization.com by one of the researchers (EB). Blocks of 4 were used. Only after the participant provided consent, the investigator opened the envelope with the allocated study group.

Due to the nature of the intervention, neither the participants nor the investigators in direct contact with the participants were blinded. Only the investigator in charge of data analysis was blinded.

### Sample Size Calculation, Data Management, and Statistical Analysis

Accepting an α risk of 0.05 and a β risk of 0.2 in a two-sided test, 31 subjects in the intervention group and 31 subjects in the control group were required to achieve a statistically significant difference ≥0.35 units in the SEMSA overall score [37]. The common SD was assumed to be 0.46 [38]. A 10% drop-rate was anticipated.

Baseline and end-of-study data (questionnaires) were collected face-to-face at the outpatient clinic by the investigators (EB, EA, and MM). Study data were collected and managed using the REDCap electronic case report form [39,40] hosted at the Hospital Clínic de Barcelona. Data on patient-reported NIV use and clinical problems with NIV were collected online using MyPathway.

Results are presented as mean (SD) or n (%). Comparisons were conducted using Chi-square or Fisher exact tests for categorical variables and Student *t* or Wilcoxon tests, depending on the distribution of the variables, for numerical variables.

### Ethics

Study approval was obtained from the Ethics Committee for Clinical Research of Hospital Clínic de Barcelona (HCB/2019/0510). Patients read, understood, and accepted informed consent, which was signed before enrolment to the study.

## Results

### Study Population

Between February and March 2019, all patients already being treated with NIV at the noninvasive ventilation clinic at the Hospital Clínic de Barcelona were assessed for eligibility. From an initial sample of 169 eligible patients, 50 (30%) did not meet the inclusion criteria, including 32 who did not have a smartphone or tablet, and 23 (14%) declined participation. Therefore, 67 patients were randomized between February and May 2019 (see the CONSORT flow diagram in Multimedia Appendix 2). One patient from the intervention group withdrew consent during the trial due to the worsening of his clinical condition. Baseline demographic and clinical characteristics are shown in Table 1 and Multimedia Appendix 3.

### Patient-Reported Outcomes

For the primary outcome, the mean SEMSA score for self-efficacy was not significantly different in the intervention group after the intervention (before: 3.4, SD 0.6; after: 3.4, SD 0.5, *P*=.51).

The perceived risks, outcome expectancies, Epworth Sleepiness Score, and EuroQol 5Q-5D questionnaire score were also not significantly different in the intervention group after the intervention (see Multimedia Appendix 3). As for the patient experience questionnaires, neither the Nijmegen Continuity Questionnaire nor the Person-Centred Coordinated Care Experience Questionnaire were statistically significantly different between the groups (see Multimedia Appendix 3).

**Table 1:** Baseline characteristics of the study groups

			Intervention (n=33)		Control (n=34)		*P* value
Age (years), mean (SD)			68 (15.8)		65 (14.7)		.31
Male gender, n (%)			19 (58)		19 (58)		>.99
Weight, mean (SD)			86 (31.6)		78 (22.4)		.15
**Educational level (n, %)**						.73	
	No schooling		3 (9)		1 (3)		
	School education		12 (36)		13 (38)		
	Professional formation		17 (52)		19 (56)		
	Doctorate or equivalent		1 (3)		1 (3)		
BMI (kg/m^2^), mean (SD)			30.5 (7.1)		28.9 (7.4)		.35
**Smoking status, n (%)**						<.001	
	Never		12 (36)		16 (49)		
	Former		18 (55)		16 (48)		
	Current		2 (6)		1 (3)		
Smoking (packs/year), mean (SD)			55.5 (35.7)		52.5 (33)		.003
**Diagnostic group, n (%)**							
	Neuromuscular		4 (12)		8 (24)		.25
	Chest wall		11 (33)		10 (30)		.81
	Obesity-hypoventilation		5 (15)		5 15)		>.99
	Airway obstructive disease		3 (9)		2 (6)		.66
	OSA^a^ to CSA^b^		10 (30)		8 (24)		.60
Number of comorbidities per patient, mean (SD)			2 (1.5)		1.8 (1.6)		.68
**Comorbidities, %**							
	Cancer		3		3		>.99
	Congestive heart disease		33		27		.60
	Ischemic heart disease		24		15		.37
	Diabetes		27		36		.47
	Stroke		9		9		>.99
	Hypertension		67		52		.20
	Dementia		3		0		.32
	Neurological disorders other than stroke		3		0		.32
	Depression/anxiety		18		18		>.99
	Dyslipidemia		15		27		.54
Time on noninvasive ventilation (years), mean (SD)			6.75 (6.5)		4.5 (3.5)		.08
AHI^c^, mean (SD)			46 (28.8)		35 (31.6)		.37
CT90^d^ (%), mean (SD)			47 (37.3)		44 (40.4)		.91
**Mean ventilatory parameters, mean (SD)**							
	IPAP^e^ (cm H_2_O)		16 (4.7)		14 (4.7)		.06
	EPAP^f^ (cm H_2_O)		7 (2.8)		6 (2.1)		.31
	Leak (L/s)		0.05 (0.2)		0.5 (0.09)		.03
	Number of hours used per day		7.4 (2)		6.8 (3)		.28

^a^OSA: obstructive sleep apnea.

^b^CSA: central sleep apnea.

^c^AHI: global apnea-hypopnea index for all diagnostic groups.

^d^CT90: cumulative sleep time percentage with oxyhemoglobin saturation <90%.

^e^IPAP: inspiratory positive airway pressure.

^f^EPAP: expiratory positive airway pressure.

### Clinical Outcomes

Adherence was measured as the number of hours the NIV was used per day, as recorded by the ventilator. The mean adherence value was not significantly different in the intervention group after the intervention (before: 7.4 hours, SD 2 hours; after: 7.7 hours, SD 2 hours). Mean minute ventilation was the only significantly different ventilatory parameter after the 3-month intervention in the intervention group (before: 7.0 L/min, SD 2 L/min; after: 6.4 L/min, SD 2.1 L/min, *P*=.03). The remaining ventilatory parameters and weight are shown in Multimedia Appendix 3. None of the patients died during the trial.

### mHealth Tool Use, Usability, and Acceptability

The Net Promoter Score was –3 (10/33, 31% promoters; 11/33, 34% passives; 11/33, 34% detractors). The 3 Likert-scale questions about the general satisfaction with the app that were rated from 1 (very bad) to 10 (very good) resulted in a mean score of 7.5/10 for the general impression of the app, mean score of 8.2/10 for the user friendliness, and mean score of 8.5/10 for usability of the app without assistance. The mean System Usability Scale score was 78, a reasonably good grading. Up to 42% of the participants used the link to the educational material, and only 18% (6/33) consulted the terms of use. The mean number of hours of NIV use per day, reported using the mHealth tool, was 7.23 hours (SD 2.48 hours). Use of NIV for more than 4 hours per day during two-thirds of the study period was reported by 45% (15/33) of the patients. Likewise, the reported mean number of days during which NIV was used more than 4 hours in the entire intervention group was 35.6 days (SD 23.6 days). At the end of the study period, 3 participants stopped reporting due to app problems, 1 participant stopped using the app due to health problems, another participant stopped using the app for unknown reasons, and 3 participants decided to use the app on an alternative day basis.

Also, 30% (10/33) of the participants used the app through a family member or carer. It is of note that the nurse case manager was able to solve two-thirds of the technical problems that arose during the first 3 weeks of the study.

The qualitative analysis of the motivational interview as well as the detailed description of the requirements for mHealth to support collaborative work among stakeholders will be reported elsewhere. However, Table 2 summarizes a list of features that the research team agreed were key functional requirements of mHealth tools to effectively support collaborative work among stakeholders involved in home-based respiratory therapies.

**Table 2:** Requirements to support collaborative work within the noninvasive ventilation service.

Feature	Description of the requirement(s)
Adaptive case management	Capacity to enable the case manager to combine predesigned tasks and approach new cases by reusing structured experiences with previous cases. Over time, the case manager, or other authorized health professionals, should be able to adapt the work plan in a timely fashion to specific patient’s requirements without any direct technological support
Team collaboration	Cloud-based, General Data Protection Regulation-compliant, enterprise-proven team collaboration tools to allow patients and health care professionals to break down silos and collaborate seamlessly from any device (mobile phone, tablet, or desktop) towards the health continuum care pathway
Multimedia communication	Enterprise-grade, scalable, high-quality, real-time communication among concurrent participants for file sharing, voice, video, and screen-share sessions with industry-standard encryption
Intelligent bots	Capacity to develop and integrate intelligent bots to guide professionals through continuum care pathways and to improve health risk assessment and service selection
Integration with hospital information systems	Use of HL7 Fast Healthcare Interoperability Resource interoperable middleware to integrate with provider-specific hospital information systems

## Discussion

### Principal Findings for Patient-Reported Outcomes

We report the results of a behavioral mHealth intervention based on a face-to-face interview and the use of an mHealth tool (MyPathway app) during a 3-month follow-up period with patients with hypercapnic chronic respiratory failure under home-based long-term NIV. To the best of our knowledge, this is the first randomized controlled trial using digital tools to support behavioral changes in this population [41-44].

In this study, the mean self-efficacy score was already high at baseline (Table 1), and we did not find a significant effect of the intervention on behavioral changes. Several explanations can be proposed for these results. First, the intervention may need to be more intensive (ie, more than one face-to-face session) [45]. Second, all the participating patients were long-term users without significant sleep symptoms at the time of enrollment (average use >6 years with an average Epworth Sleepiness Score <10). Therefore, we could hypothesize that behavioral changes had occurred previously, as evidenced by the good average use of NIV (7.4 h/day) and high scores for self-efficacy at baseline. The inclusion of patients who have been newly prescribed NIV in future studies may show a positive impact of the intervention. Third, we may argue that, although NIV use was good among this sample of long-term users, adherence was more a function of necessity or imposition (by family or physicians) than a real feeling of self-management and that most of these chronic patients had not considered initiating behavioral changes [46,47]. Along this line of thought, the population we studied had mobility problems or poor general health, creating barriers for behavioral change [20]. Therefore, any intervention at this stage is likely to be ineffective. This may also be reflected by the lack of interest in consulting the educational material in the app (<50% of the patients did so). Last, we should note that the control group consisted of more patients with neuromuscular pathophysiology. However, the pathophysiology should not affect or have a direct relationship with the measured behavioral outcomes or the capacity and readiness to use the app. Accordingly, educational level is a more important factor [48,49], and both study groups had similar educational levels.

### Usability, Acceptability, and Requirements for Supporting Collaborative Work

Notwithstanding the clinical results, it is important to note that the mHealth tool was well received by the patients and their family/caregivers. Despite their complex conditions (2 comorbidities on average) with considerable needs and burdensome treatment, all patients used the app regularly, grading it as generally good, user-friendly, and easy to use without help. Moreover, the System Usability Scale score was good.

As stated in the methods section, we want to highlight the fact that one of the authors (MM) undertook a new professional role during the study period. She became the clinical case manager with additional technical knowledge on the mHealth tool. Patients appreciated this new role very much despite the use of telephone or Whatsapp for bilateral communication. We found that the app lacked this function, and based on our experience, this should become an integral part of any app that includes case management with technical skills. This type of communication functionality should be cloud-based and General Data Protection Regulation-compliant. Moreover, future developments should consider adaptive case management functionality. Also, this communication should be supported by artificial intelligence to help guide professionals though continuum care pathways and improve health risk assessment and service selection. Finally, integration with hospital information systems may facilitate the whole process. This is in line with a recent report on the digital transformation of health care in Europe, which draws upon the experiences of 17 integrated care programs where the importance of communication technologies, new professional roles, and the relevance of clinical workflow evaluation were highlighted [50].

In this respect, we measured 2 process outcomes [51] related to patient experience [52]: continuity of care and person-centered care. Our study population, which included patients as well as their family and carers for one-third of the cases in the intervention group, evaluated both parameters very well. The importance of well-designed clinical workflows with embedded digital health tools may have an impact on not only an NIV service but also other respiratory services. Commonalities include high-complexity patients with clinical and social needs from different stakeholders (eg, physicians, providers, technicians, social workers) and health care tiers (eg, primary care, specialized care). Hernandez et al [24] showed how this complexity can hamper the effectiveness of long-term oxygen therapy. As mentioned, Table 2 shows the proposed elements to overcome the barriers for the successful implementation of digital health tools within clinical workflows relating to respiratory therapies.

Finally, stakeholders play an important role in the design and evaluation of digital health tools [53,54] and, as such, their input should be taken into account when evaluating a service in which there is considerable interplay between patients, different health care tiers, and social and technical services [55]. For an mHealth tool to produce health care value, it should be embedded in the clinical pathways of a well-evaluated clinical service and not as a standalone tool [56].

### Strengths and Limitations of the Study

Our study considered the whole population of patients attending the clinic, resulting in a realistic clinical scenario. Another important strength of our study is its potential to demonstrate the positive interaction and collaborative work among the nurse case manager, patients, and family members or caregivers of complex patients using digital health tools. Previous studies [57-59] reported the use of digital tools by family caregivers, emphasizing the importance of including this group of stakeholders, not only as users but also in the co-design process. This stakeholder involvement is also a further step in scaling up digital health tools within clinical workflows [59], which, in our case, were evaluated well. An interesting aspect of our study was the collateral use of qualitative data collected from the motivational interviews and by the nurse case manager during follow-up. The qualitative results presented in Table 2 can be used to support the implementation of mHealth tools in different contexts, keeping in mind the inherent limitations of qualitative research data. We do acknowledge that, by using an existing app, the co-design phase was skipped. Also, we did not measure the technological literacy of our older population (average age 69 years), but, according to Martinez-Alcala et al [60], adults older than 60 years, if highly motivated, are capable of learning and acquiring digital literacy skills. Nonetheless, for some of our older patients (24% were 70-79 years old), especially those with physical limitations (eg, visual impairment), the motivation to learn and exploit all the app functionality was low, although the perceived usefulness was high. This agrees with other reports on the use of technology by older adults [61,62]. Another potential limitation was the heterogeneity of the study population, which directly influenced the mean number of hours of use of the NIV machines and precludes any interpretation. Nonetheless, we observe a strength in terms of the generalizability of the mHealth tools within the heterogeneous population. Finally, a clear limitation of our study was the exclusion of new NIV patients, where the behavioral intervention may have had more impact. This warrants further study.

### Conclusions

The behavioral mHealth intervention explored in this study did not show any effect on self-efficacy, adherence with NIV, or quality of life in our population of experienced NIV users. Nonetheless, we showed the potential of the mHealth app to manage complex patients and foster collaborative work among stakeholders. Regarding a clinical service that was graded well in terms of continuity of care and person-centered care, in which the needs of relevant stakeholders are properly addressed, we see the potential to further study mHealth tools to induce behavioral change in home-based ventilated patients as well as in other respiratory therapies.

## Appendix

Multimedia Appendix 1 Mypathway adaptation for home-based non-invasive ventilation.

Multimedia Appendix 2 Consort flow diagram.

Multimedia Appendix 3 Baseline characteristics and clinical outcomes tables.

Multimedia Appendix 4 CONSORT-eHEALTH checklist (V 1.6.2).

## Abbreviations

ICT: information and communication technologies
mHealth: mobile health
NIV: noninvasive ventilation
SEMSA: Self Efficacy in Sleep Apnea
