# Supplementary material for: Integrated Care Intervention Supported by a Mobile Health Tool for Patients Using Noninvasive Ventilation at Home: Randomized Controlled Trial
Source: JMIR Mhealth Uhealth. 2020 Apr 13;8(4):e16395. doi: 10.2196/16395 (PMC7186864; doi:10.2196/16395)
Supplement: Multimedia Appendix 1 [file mhealth_v8i4e16395_app1.docx]

## Multimedia Appendix 1: MyPathway adaptation for home-based non-invasive ventilation

**Objective:** Customize MyPathway for Home-based non-invasive ventilation (NIV) and integrate MyPathway with the health information systems of Hospital Clínic (with a HL7-FHIR integration middleware).

As depicted in **Figure 1**, the NIV service considered as key supporting technologies an adaptive case management platform to enhance collaborative work among health professionals and patients themselves using a personal health system for patient self-management at community level with remote capture of patient reported outcomes (PROMs) to answer study questionnaires, delivery of general educational material and personalized recommendations and monitoring of self-reported hours of use (daily). Most importantly, these key supporting technologies were required to be ready for integration with Hospital Clínic information systems (i.e. SAP) and the regional health information systems for a large scale development in the region (i.e., Catalonia).


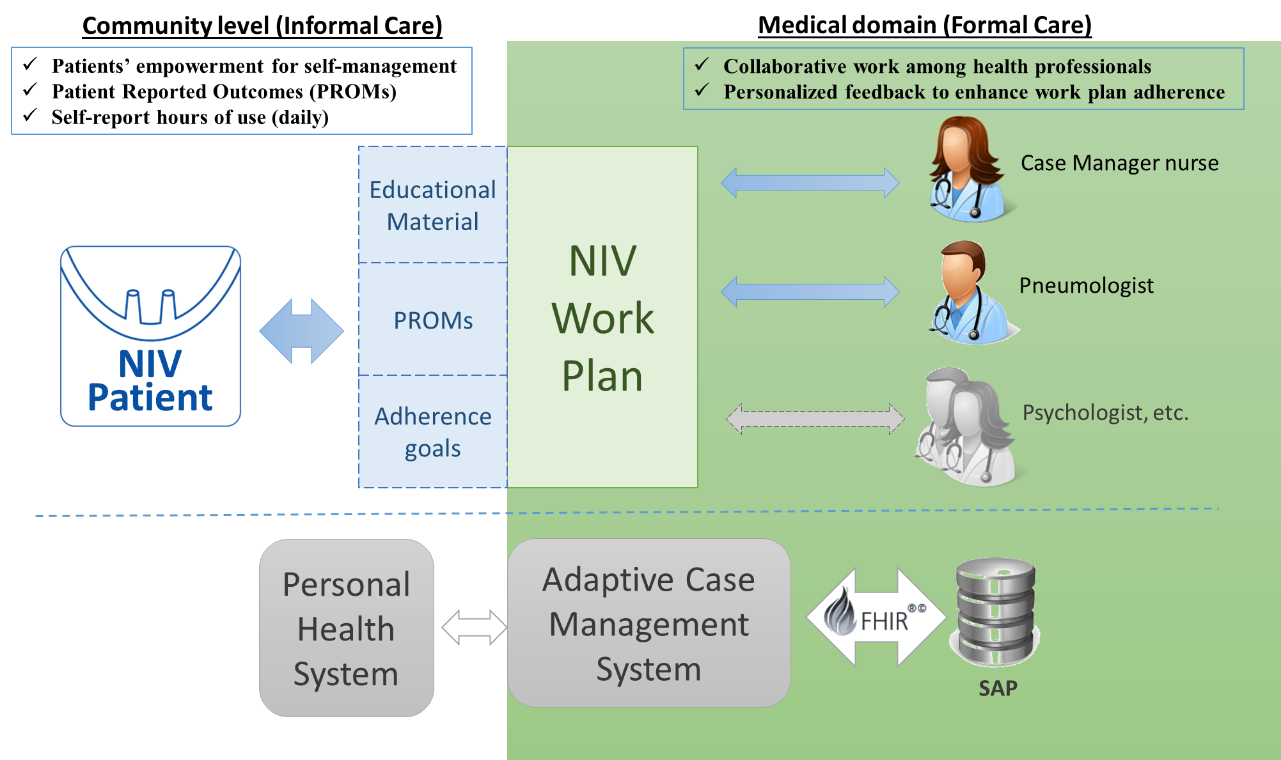


*Figure 1 - The figure shows two interoperable domains with technological elements providing support to NIV. On the left hand side, the Informal Care area considers the patient access to the NIV Personal Health System wherein she/he can answer study questionnaires (PROMs), report hours of daily use and have access to tailored educational information, as defined in the NIV work plan (centre of the figure). On the right hand side, the Formal Care domain includes the NIV team (Case manager nurse, pneumologist, Psychologist, etc.), with access to an adaptive case management system for work plan prescription, follow-up and coaching. The adaptive case management system supports execution of the patient work plan and provides a bridge of interoperability and collaborative tools among the patient (through the NIV personal health system), the NIV team and the electronic medical record (i.e. SAP in case of Hospital Clínic).*

For the design, technical deployment and integration of the supporting technologies in a stepwise manner, the NIV service prioritised the implementation of the personal health system for patient self-management at community level. Specifically, a personal health system (MyPathway®) currently being adapted in hospitals in the UK (e.g., Sheffield Teaching Hospital) was adapted. MyPathway® is a secure, digital communications channel connecting patients to clinicians and services. It is a browser and app-based application co-designed and tested by users to make it user-friendly for both patients and clinicians to use on phones, tablets and PCs.

Although MyPathway® already allowed for automated PROMs capture, such as EQ-5D™, Oxford Hip Score and MSK-HQ, the NIV service had different requirements (**Table 1**), including the use of specific questionnaires to report predefined clinical problems: i) dry mouth, ii) red eyes, iii) mask noise, iv) mask leak sensation, v) diurnal somnolence and vi) weight gain. In addition, since a key requirement of the NIV service is the capacity to prescribe and remotely self-report daily use of NIV, MyPathway was extended to allow for manual prescription of daily NIV use goals.

| **Feature** | **Description** |
| --- | --- |
| **Spanish and Catalan languages** | Hospital Clínic facilitates translation to Spanish and Catalan both for the clinician’s portal and the patient’s web/app |
| **Monitoring of patient’s daily use of NIV** | Patient-specific target daily use of NIV (i.e., number of daily hours) will be prescribed by healthcare professionals (number of target daily hours should be customisable dynamically and the prescription could be cancelled anytime.). Patients will receive the prescription in MyPathway timeline (in the form of a daily goal), to be manually answered by the patient. Based on self-reported hours of use of NIV, motivational feedback will prompt the patient to continue in the same line or try change his/her behaviour by identifying any of the specific problems mentioned below. |
| **PROMs** | Periodically (weekly or when the patient self-report less than 4 hours of daily use), MyPathway will use specific questionnaires to report predefined clinical problems: i) dry mouth, ii) red eyes, iii) mask noise, iv) mask leak sensation, v) diurnal somnolence and vi) weight gain. |
| **Integration with hospital information systems** | Patient referral to the NIV program will trigger the creation of a new user in the clinician’s portal and will send the invitation to the patient for registering to MyPathway. Acceptance of the invitation will trigger the allocation of the on-boarding material to the patient timeline. |

Table 1 – Adaptation requirements of MyPathway to support the NIV service.

**Figure 2** below illustrates with screenshots the main functionality of the NIV personal health system.


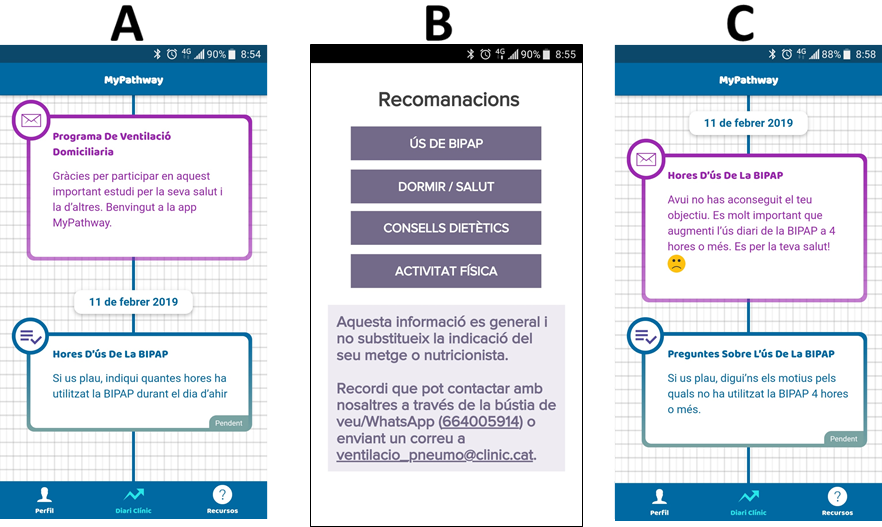


*Figure 2 – Look-and-feel of main functionalities of NIV personal health system: Welcome message (Panel A – purple timeline message) with link to on-boarding material and daily NIV use goal (Panel A – blue timeline message), educational material (Panel B), and tracking of goal progress with motivational feedback and self-administered PROMs (Panel C –blue timeline messages).*

Finally, the PREHAB personal health system was ready to be integrated with SAP via ORM messaging triggered by the eventual inclusion of a patient in the NIV program. The ORM message would be securely bypassed between SAP and MyPathway with a Fast Healthcare Interoperable Resource platform (HAPI FHIR) deployed in the intranet of hospital information systems. Such bypass consists on automatically sending an invitation letter to the e-mail of the patient (if already informed in the Hospital SAP) with instructions on how to access the browser and app-based version of the NIV personal health system and how to setup, for the first time, their password.
