# Supplementary figures and images for: Integrated Care Intervention Supported by a Mobile Health Tool for Patients Using Noninvasive Ventilation at Home: Randomized Controlled Trial
Source: JMIR Mhealth Uhealth. 2020 Apr 13;8(4):e16395. doi: 10.2196/16395 (PMC7186864; doi:10.2196/16395)

Multimedia Appendix 3: CONSORT flow diagram

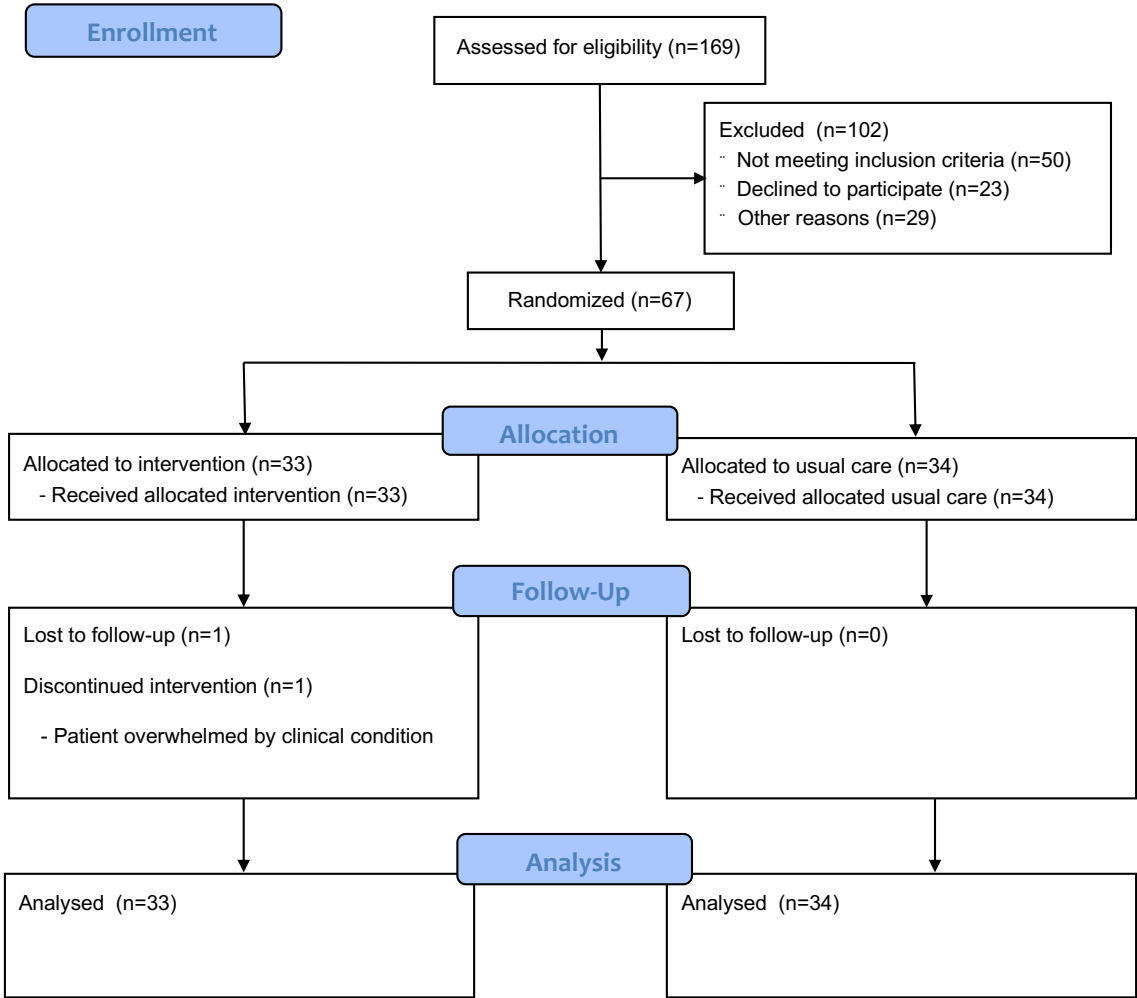

Supplement: Multimedia Appendix 2 [file mhealth_v8i4e16395_app2.pdf]
