# Supplementary material for: Integrated Care Intervention Supported by a Mobile Health Tool for Patients Using Noninvasive Ventilation at Home: Randomized Controlled Trial
Source: JMIR Mhealth Uhealth. 2020 Apr 13;8(4):e16395. doi: 10.2196/16395 (PMC7186864; doi:10.2196/16395)
Supplement: Multimedia Appendix 3 [file mhealth_v8i4e16395_app3.docx]

## Multimedia Appendix 2: Baseline characteristics and clinical outcomes tables.

## Baseline Characteristics

|  | **Intervention (n=33)** | **Control (n=34)** | ***P* values** |
| --- | --- | --- | --- |
|  |  |  |  |
| Age (mean, SD) | 68.61 (15.8) | 65 (14.7) | .31 |
| Male gender (n, %) | 19 (57.6) | 19 (57.6) | >.99 |
| Weight (mean, SD) | 86.4 (31.6) | 78 (22.4) | .15 |
| Educational level (n, %) |  |  | .73 |
| No scholarization | 3 (9.1) | 1 (2.9) |  |
| School education | 12 (36.4) | 13 (38.2) |  |
| Professional formation | 17 (51.5) | 19 (56) |  |
| Doctorate or equivalent | 1 (3) | 1 (2.9) |  |
| BMI | 30.5 (7.1) | 28.9 (7.4) | .35 |
| Smoking Status (n, %) |  |  | <.001 |
| Never | 12 (36.4) | 16 (48.5) |  |
| Past | 18 (54.5) | 16 (48.5) |  |
| Current | 2 (6.1) | 1 (3) |  |
| Pack/year (mean, SD) | 55.5 (35.7) | 52.5 (33) | .003 |
| Diagnostic group (n, %) |  |  |  |
| Neuromuscular | 4 (12) | 8 (24) | .25 |
| Chest wall | 11 (33) | 10 (30) | .81 |
| Obesity-hypoventilation | 5 (15) | 5 15) | >.99 |
| Airway obstructive disease | 3 (9) | 2 (6) | .66 |
| OSA – cOSA | 10 (30) | 8 (24) | .60 |
| Comorbidities |  |  |  |
| Number/patient (mean) | 2 | 1.8 | .68 |
| Cancer (%) | 3 | 3 | >.99 |
| Congestive heart disease (%) | 33 | 27 | .60 |
| Ischemic heart disease (%) | 24 | 15 | .37 |
| Diabetes (%) | 27 | 36 | .47 |
| Stroke (%) | 9 | 9 | >.99 |
| Hypertension (%) | 67 | 52 | .20 |
| Dementia (%) | 3 | 0 | .32 |
| Other neurological disorders (%) | 3 | 0 | .32 |
| Depression / anxiety (%) | 18 | 18 | >.99 |
| Dyslipidemia (%) | 15 | 27 | .54 |
| Time on NIV (months) (mean, SD) | 81.55 (78.3) | 54.64 (42.4) | .08 |
| AHI (mean, SD) | 45.74 (28.8) | 34.59 (31.6) | .37 |
| CT90 (%)(mean, SD) | 46.88 (37.3) | 43.88 (40.4) | .91 |
| Pulmonary Function Tests |  |  |  |
| FEV1 (%)(mean, SD) | 48.8 (19.6) | 48.1 (21.7) | .92 |
| FVC (%)(mean, SD) | 58.8 (23) | 52.4 (19.8) | .19 |
| FEV1/FVC (mean, SD) | 62 (19.6) | 71 (15.6) | .02 |
| TLC (%)(mean, SD) | 85 (20.2) | 73.3 (23.5) | .25 |
| RV (%) (mean, SD) | 122.1 (38) | 116.5 (46.5) | .36 |
| DLCO (%) (mean, SD) | 54.7 (16.2) | 55.4 (14.7) | .51 |
| KCO (%) (mean, SD) | 83.9 (17.9) | 86.9 (23.1) | .19 |
| Arterial blood gases |  |  |  |
| pH (mean, SD) | 7.40 (0.4) | 7.39 (0.2) | .26 |
| pO2 (mmHg) (mean, SD) | 75.2 (17.9) | 75.3 (11.1) | .82 |
| pCO2 (mmHg) (mean, SD) | 44.7 (8) | 45.2 (7.4) | .92 |
| HCO3 (mEq/L) (mean, SD) | 27.4 (4.2) | 27 (3.8) | .40 |
| Mean ventilatory parameters |  |  |  |
| IPAP (cmH2O) (mean, SD) | 16.3 (4.7) | 14.1 (4.7) | .06 |
| EPAP (cmH2O) (mean, SD) | 7 (2.8) | 6.3 (2.1) | .31 |
| Vmin (L/min) (mean, SD) | 7.3 (2) | 6.8 (2.4) | .55 |
| Tidal volume (ml) (mean, SD) | 411(128) | 358 (148) | 0.77 |
| Back-up frequency (breaths/min) (mean, SD) | 17 (3) | 16 (3) | .65 |
| Leak (L/s) (mean, SD) | 0.05 (0.2) | 0.5 (0.09) | .03 |
| N° of hours use/day (mean, SD) | 7.5 (2) | 6.8 (3) | .28 |
| NIV interface (n, %) |  |  | >.99 |
| Nasal | 13 (39) | 13 (39) |  |
| Full-face | 20 (61) | 20 (61) |  |
| Traqueostomy | 0 | 0 |  |

### Patient reported outcomes

a) Task self-efficacy, sleepiness and quality of life questionnaires

|  | Control (n=34) | | | Intervention (n=33) | | |
| --- | --- | --- | --- | --- | --- | --- |
|  | Baseline | Post 3-monts | *P* | Baseline | Post 3-monts | *P* |
| SEMSA Perceived risks | 1.8 (0.7) | 1.7 (0.7) | .22 | 1.8 (0.7) | 1.6 (0.5) | .06 |
| SEMSA Outcome expectancies | 2.9 (0.6) | 3 (0.5) | .62 | 2.8 (0.6) | 2.9 (0.6) | .33 |
| SEMSA Self-efficacy | 3.4 (0.4) | 3.5 (0.4) | .43 | 3.4 (0.6) | 3.4 (0.5) | .51 |
| ESS | 8 (5.8) | 7.7 (6) | .69 | 6.6 (5.6) | 7.2 (6.3) | .28 |
| EQ-5D index value | 0.76 (0.13) | 0.77 (0.14) | .38 | 0.79 (0.14) | 0.8 (0.12) | .54 |
| EQ-5D VAS | 59.2 (22) | 58.3 (28.5) | .83 | 60.7 (19) | 65.7 (21.5) | .19 |

Data are mean (standard deviation); SEMSA: Self-efficacy measure for sleep apnea; ESS: Epworth sleepiness score; EQ-5D: EuroQol 5D

b) Patient experience questionnaires

|  | Control (n=34) | Intervention (n=33) | *P* |
| --- | --- | --- | --- |
|  | Post 3-months | Post 3-months |  |
| NCQ | 3.9 (0.9) | 4.1 (0.7) | .42 |
| P3CEQ | 14.1 (3.6) | 15.4 (2.9) | .12 |

NCQ: Nijmegen continuity of care questionnaire; P3CEQ: Person centred coordinated experience questionnaire

### Clinical outcomes

|  | Control (n=34) | | | Intervention (n=33) | | |
| --- | --- | --- | --- | --- | --- | --- |
|  | Baseline | Post 3-monts | *P* | Baseline | Post 3-monts | *P* |
| IPAP (mmH2O) | 13.9 (4.7) | 14.5 (4.3) | **.02** | 16.3 (4.7) | 16.6 (3.8) | .64 |
| EPAP (mmH2O) | 6.2 (1.9) | 6.7 (2.2) | **.03** | 7 (2.8) | 7.3 (2.3) | .45 |
| Vmin (L/min) | 6.6 (2.5) | 6.5 (2.6) | .92 | 7 (2) | 6.4 (2.1) | .**03** |
| Tidal volume (ml) | 381.6 (133.8) | 378.4 (133.8) | .70 | 407.2 (132.7) | 370.6 (140.6) | .04 |
| Breaths/min | 16.6 (3.5) | 16.3 (3.7) | .48 | 16.5 (3.4) | 16.3 (2.4) | .65 |
| Leak (L/s) | 0.05 (0.09) | 0.1 (0.14) | .**01** | 0.05 (0.19) | 0.14 (0.16) | .07 |
| NIV use (h/day) | 6.8 (2.6) | 7.2 (2.3) | .20 | 7.4 (2) | 7.7 (2) | .28 |
| Weight (kg) | 78.8 (22.4) | 78.7 (23) | .88 | 85.6 (31.8) | 83.8 (28) | .56 |

Data are mean (standard deviation); IPAP: Inspiratory positive airway pressure; EPAP: Expiratory positive airway pressure; Vmin: minute ventilation; NIV: noninvasive ventilation

### NIV usage reported by patients using MyPathway®

| **App usage reported by patients** | **N or %** |
| --- | --- |
| N° of hours of reported use/day for all patients (mean ±SD) | 7.23 ±2.48 |
| NIV reported use ≥ 4 hours a day, during 2/3 of days (% of patients) | 44.9 |
| N° of days of NIV reported use ≥ 4 hours (mean ±SD) | 35.67 ±23.63 |
| N°of reasons reported by all patients that used BIPAP < 4 hours a day (mean) | 1.4 |
| Leak sensation around the mask (%) | 8.57 |
| Ocular irritation (%) | 31.43 |
| Dry mouth (%) | 25.71 |
| Unable to sleep with NIV (%) | 20 |
| Unable to put the mask / turn-on the machine (%) | 28.57 |
| Don’t feel like using the machine (%) | 25.71 |
| N° of weekly symptoms/problems reported by all patients that used BIPAP < 4 hours a day, during 2/3 of days (mean) | 1.92 |
| Leak sensation around the mask (%) | 18.82 |
| Ocular irritation (%) | 34.12 |
| Mask noise (%) | 28.24 |
| Excessive sleepiness (%) | 16.47 |
| Dry mouth / cold air sensation in the mouth (%) | 45.88 |
| Facial irritation (%) | 28.24 |
| N° of weekly symptoms/problems reported by all patients that used BIPAP ≥ 4 hours a day, during 2/3 of days (mean) | 1.72 |
| Leak sensation around the mask (%) | 27.22 |
| Conjunctival erythema (%) | 18.94 |
| Mask noise (%) | 27.81 |
| Excessive sleepiness (%) | 23.67 |
| Dry mouth / cold air sensation in the mouth (%) | 56.81 |
| Facial irritation (%) | 17.16 |

### MyPathway® logobook analysis

| **Dispositive** | | | | | **Total Participants** | | | | | | | | | | **Total**  **(%)** | | | | | | | |
| --- | --- | --- | --- | --- | --- | --- | --- | --- | --- | --- | --- | --- | --- | --- | --- | --- | --- | --- | --- | --- | --- | --- |
| Smartphone | | | | | 32 | | | | | | | | | | 96,97 | | | | | | | |
| Tablet | | | | | 1 | | | | | | | | | | 3,03 | | | | | | | |
| **Total** | | | | | **33** | | | | | | | | | | **100,00** | | | | | | | |
| **Operative System** | | | | | **Total Participants** | | | | | | | | | | **Total**  **(%)** | | | | | | | |
| Android | | | | | 27 | | | | | | | | | | 81,82 | | | | | | | |
| iOS | | | | | 6 | | | | | | | | | | 18,18 | | | | | | | |
| **Total** | | | | | **33** | | | | | | | | | | **100,00** | | | | | | | |
| **Who registered data in th app** | | | | | **Total Participants** | | | | | | | | | | **Total**  **(%)** | | | | | | | |
| Daughter or son | | | | | 7 | | | | | | | | | | 21,21 | | | | | | | |
| Pacient | | | | | 23 | | | | | | | | | | 69,70 | | | | | | | |
| Partner | | | | | 2 | | | | | | | | | | 6,06 | | | | | | | |
| Other family member | | | | | 1 | | | | | | | | | | 3,03 | | | | | | | |
| **Total** | | | | | **33** | | | | | | | | | | **100,00** | | | | | | | |
| **SMS useful** | | | | | **Total Participants** | | | | | | | | | | **Total**  **(%)** | | | | | | | |
| No | | | | | 5 | | | | | | | | | | 15,15 | | | | | | | |
| Yes | | | | | 19 | | | | | | | | | | 57,58 | | | | | | | |
| No information | | | | | 9 | | | | | | | | | | 27,27 | | | | | | | |
| **Total** | | | | | **33** | | | | | | | | | | **100,00** | | | | | | | |
| **SMS perception** | **Total** | | | | | | | | | | | | | | | | | | | | | |
| Bothersome | 1 | | | | | | | | | | | | | | | | | | | | | |
| Received by other family member’s phone | 1 | | | | | | | | | | | | | | | | | | | | | |
| Receives it late | 1 | | | | | | | | | | | | | | | | | | | | | |
| No information | 1 | | | | | | | | | | | | | | | | | | | | | |
| Prefers Whatsapp® | 1 | | | | | | | | | | | | | | | | | | | | | |
| **Total** | **5** | | | | | | | | | | | | | | | | | | | | | |
| **Data registry using the app** | | **Study Week** | | | | | | | | | | | | | | **Total** | | | | **%** | | |
|  |  | **1** | **2** | **3** | | **4** | **5** | **6** | | | **8** | | **9** | | |  | | | |  | | |
| Stopped registry due to app problems | | 7 | 2 |  | |  |  | 3 | | |  | |  | | | **12** | | | | **4,55** | | |
| Stopped registry due to health problems | | 1 |  |  | |  |  | 1 | | | 1 | |  | | | **3** | | | | **1,14** | | |
| Did not register | | 2 | 3 | 5 | | 3 | 3 | 3 | | | 3 | | 1 | | | **23** | | | | **8,71** | | |
| Registry for 5-6 days | |  | 13 | 6 | | 13 | 9 | 8 | | | 9 | | 7 | | | **65** | | | | **24,62** | | |
| Sporadic registry | |  |  |  | | 1 |  |  | | |  | |  | | | **1** | | | | **0,38** | | |
| Alternate days registry | | 3 | 1 | 7 | | 6 | 5 | 6 | | | 6 | | 3 | | | **37** | | | | **14,02** | | |
| Everyday registry | | 20 | 14 | 15 | | 10 | 8 | 3 | | | 4 | |  | | | **74** | | | | **28,03** | | |
| Complete all the registry | |  |  |  | |  | 8 | 9 | | | 10 | | 22 | | | **49** | | | | **18,56** | | |
| **Total** | | **33** | **33** | **33** | | **33** | **33** | **33** | | | **33** | | **33** | | | **264** | | | | **100** | | |
| **Incidences** | | | | | | | | | **Study week** | | | | | | | | | |  | | |  |
| **Type of incidence** | | | | | | | | | **1** | | | | | **2** | | | **3** | | **Total** | | | **(%)** |
| The data record in the app is not reflected in the platform | | | | | | | | | 1 | | | | | 1 | | | 2 | | **4** | | | **14,81** |
| The app asks for logging every time | | | | | | | | | 1 | | | | |  | | |  | | **1** | | | **3,70** |
| Cannot fill weekly questionnaire due to lack of options | | | | | | | | | 1 | | | | |  | | |  | | **1** | | | **3,70** |
| Password problems | | | | | | | | |  | | | | |  | | | 1 | | **1** | | | **3,70** |
| Always have to login with username and password | | | | | | | | | 2 | | | | | 1 | | | 1 | | **4** | | | **14,81** |
| White screen | | | | | | | | | 11 | | | | |  | | | 1 | | **12** | | | **44,44** |
| Accidentally logout and forgot login details | | | | | | | | |  | | | | |  | | | 1 | | **1** | | | **3,70** |
| Incidence without details (participant could not be contacted) | | | | | | | | | 1 | | | | | 1 | | | 1 | | **3** | | | **11,11** |
| **Total** | | | | | | | | | **17** | | | | | **3** | | | **7** | | **27** | | | **100** |
| **Incidences solution** | | | | | | | | | | **Study week** | | | | | | | | | | | | |
|  | | | | | | | | | | **1** | | **2** | | | **3** | | | **Total** | | | **(%)** | |
| App problem not solved at first contact | | | | | | | | | | 6 | | 2 | | | 3 | | | 11 | | | 40,74 | |
| App problem not solved (participant abandoned the study) | | | | | | | | | | 1 | |  | | |  | | | 1 | | | 3,70 | |
| App problem not solved (participant cannot be contacted) | | | | | | | | | | 1 | | 1 | | | 1 | | | 3 | | | 11,11 | |
| App problem solved at first contact | | | | | | | | | | 9 | |  | | | 3 | | | 12 | | | 44,44 | |
| **Total** | | | | | | | | | | **17** | | **3** | | | **7** | | | **27** | | | **100** | |
