# Supplementary material for: Integrated Care Intervention Supported by a Mobile Health Tool for Patients Using Noninvasive Ventilation at Home: Randomized Controlled Trial
Source: JMIR Mhealth Uhealth. 2020 Apr 13;8(4):e16395. doi: 10.2196/16395 (PMC7186864; doi:10.2196/16395)
Supplement: Multimedia Appendix 4 [file mhealth_v8i4e16395_app4.pdf]

CONSORT-EHEALTH Checklist V1.6.2 Report	Manuscript Number	16395
**(based on CONSORT-EHEALTH V1.6), available at [http://tinyurl.com/consort-ehealth-v1-6].**		
**Date completed**		
9/26/2019 2:33:24		
**by**		
Erik Baltaxe		
An Integrated Care Intervention Supported by a Mobile Health Tool in Patients Using Noninvasive Ventilation at Home: Randomized Controlled Trial		
TITLE		
**1a-i) Identify the mode of delivery in the title**		
"Mobile Health Tool"		
**1a-ii) Non-web-based components or important co-interventions in title**		
"Integrated care intervention"		
**1a-iii) Primary condition or target group in the title**		
"in Patients Using Noninvasive Ventilation at Home"		
ABSTRACT		
**1b-i) Key features/functionalities/components of the intervention and comparator in the METHODS section of the ABSTRACT**		
"A single blinded, single center, randomized controlled trial was performed on 67 adult patients with chronic respiratory failure undergoing home-based noninvasive ventilation, between February and June 2019. In the intervention group, a psychologist delivered a face-to-face motivational intervention. Follow-up was supported by a mobile app, which allowed patients to report the number of hours of daily use and problems with the therapy. Advice was automatically delivered by the mobile app in case a problem was reported. The control group received usual care."		
**1b-ii) Level of human involvement in the METHODS section of the ABSTRACT**		
**1b-iii) Open vs. closed, web-based (self-assessment) vs. face-to-face assessments in the METHODS section of the ABSTRACT**		
**1b-iv) RESULTS section in abstract must contain use data**		
**1b-v) CONCLUSIONS/DISCUSSION in abstract for negative trials**		
INTRODUCTION		
**2a-i) Problem and the type of system/solution**		
"Despite its proven cost-effectiveness, patients' adherence to home-based NIV has still potential to improve, which should further enhance healthcare efficiencies of the intervention". "Nevertheless, improvement of behavioral aspects such as patient motivation and empowerment for self-management are important factors to consider when addressing adherence to respiratory therapies."."The current study seeks to explore the transfer of previous positive experiences on behavioral interventions in other fields (i.e. physical activity) into home-based NIV. Specifically, we will address the concept of self-efficacy,"."We propose the use of a behavioral mHealth intervention to support changes in self-efficacy,". "information and communication technologies (ICT) have been identified as promising to generate efficiencies by enhancing coordination between stakeholders and contributing to improve health outcomes [26, 27]. Nonetheless, it is acknowledged that the scenario is not still mature [28]. Mainly, because of lacking evidence in real-world scenarios for the capacity of ICT to escort behavioral changes including self-efficacy in chronic complex patients."."The principal objective of the current study is to explore the capacity of a behavioral mHealth intervention to increase patient empowerment for self-management and adherence to therapy; whereas, the secondary aim is to learn, based on professionals' and patients' experience, how the mHealth tool should evolve to support collaborative work."		
**2a-ii) Scientific background, rationale: What is known about the (type of) system**		
" Besides self-efficacy as a way to influence behavioral change, previous reports by Hernandez et al [24] and Cano et al [25] have identified two commonalities usually hindering effective implementation of complex respiratory therapies (i.e. long-term oxygen therapy, continuous positive airway pressure therapy, home NIV and home-based nebulizer therapy). Firstly, is the need for interaction and communication among several stakeholders, namely: health professionals at different healthcare tiers (primary care, specialized care, etc.), patients and carers, companies undertaking maintenance of the equipment, and others, which may greatly benefit from digital tools supporting collaborative work. Secondly, is the improvement in therapeutic adherence that should be achieved through patients' empowerment for self-management. "		
**Does your paper address CONSORT subitem 2b?**		
"The principal objective of the current study is to explore the capacity of a behavioral mHealth intervention to increase patient empowerment for self-management and adherence to therapy; whereas, the secondary aim is to learn, based on professionals' and patients' experience, how the mHealth tool should evolve to support collaborative work."		
METHODS		
**3a) CONSORT: Description of trial design (such as parallel, factorial) including allocation ratio**		
"A single-blinded, single center, randomized controlled trial with two parallel arms (1:1 ratio) was conducted"		
**3b) CONSORT: Important changes to methods after trial commencement (such as eligibility criteria), with reasons**		
There were no changes in the methodology once the study started		
**3b-i) Bug fixes, Downtimes, Content Changes**		
**4a) CONSORT: Eligibility criteria for participants**		
"Inclusion criteria were defined as follows: all adult patients with hypercapnic ventilatory failure due to chest wall, neuromuscular, lung parenchyma and/ or airway disease already receiving treatment with NIV irrespective of treatment duration and having a mobile phone, tablet or personal computer that could support the use of the mobile health application (MyPathway®). MyPathway® [29] is a secure, digital communications channel connecting patients to clinicians and services. It is an app-based application for both patients and clinicians to use on phones and/or tablets. See Multimedia Appendix 1 for more details. Patients with severe psychiatric and/or neurological diseases where excluded, as well as those hospitalized at time of assessment. "		
**4a-i) Computer / Internet literacy**		
**4a-ii) Open vs. closed, web-based vs. face-to-face assessments:**		
"All the eligible patients were contacted by telephone in order to briefly explain the study and invite them to participate. Those showing interest were invited to the hospital outpatient clinics. Study investigators (EB, EA and MM) explained the study face-to-face and in case of acceptance, consent was signed".		
**4a-iii) Information giving during recruitment**		
**4b) CONSORT: Settings and locations where the data were collected**		
"At time of enrollment, semi-structured motivational interviews were conducted individually. Participants were asked about the following topics: (i) Treatment adaptation experience, (ii) Lifestyle (physical activity and food habits) and (iii) Use of information and communication technologies. In each session, field notes were taken anonymously, and no recordings were made. The intervention consisted of a 10 to 50-minutes face-to-face session at the hospital or at the participants' home". "During follow-up, the MyPathway® app was used by study participants for bi-directional interaction with the research team. It consisted of positive feedback or reinforcement messages in response to the number of hours of NIV use filled by the patient in a daily basis.". "A web-based clinical portal enabled the research team to monitor the NIV hours of use and clinical problems reported by the patients". "a dedicated nurse (one of the authors, MM) took the role of case manager, with clinical and technical knowledge, in order to support collaborative work. She used the web-based portal to identify adherence problems and, accordingly, she contacted the participants via telephone or at home (for those with severe mobility problems) in order to enquire about potential problems, either clinical or technical and solve them."		
**4b-i) Report if outcomes were (self-)assessed through online questionnaires**		
"All assessments were done at baseline and at three months in the final visit programmed in the outpatient clinic for the control group. For the intervention group, the follow-up was done remotely, by the nurse case manager (MM), using the MyPathway app® and its clinical portal. When deemed necessary, the nurse case manager visited the patient at home or a visit was programmed at the outpatient clinics. There was no active follow-up for the control group."		
**4b-ii) Report how institutional affiliations are displayed**		
**5) CONSORT: Describe the interventions for each group with sufficient details to allow replication, including how and when they were actually administered**		
**5-i) Mention names, credential, affiliations of the developers, sponsors, and owners**		
**5-ii) Describe the history/development process**		
**5-iii) Revisions and updating**		
**5-iv) Quality assurance methods**		

**5-v) Ensure replicability by publishing the source code, and/or providing screenshots/screen-capture video, and/or providing flowcharts of the algorithms used**		
**5-vi) Digital preservation**		
**5-vii) Access**		
"Free access was granted after receiving an invitation via the hospital Health Information System (SAP®), which prompted the participant to register using an email address as a username. The application could also be downloaded to the carers' phone in case the patient did not have a smartphone "		
**5-viii) Mode of delivery, features/functionalities/components of the intervention and comparator, and the theoretical framework**		
"At time of enrollment, semi-structured motivational interviews were conducted individually. Participants were asked about the following topics: (i) Treatment adaptation experience, (ii) Lifestyle (physical activity and food habits) and (iii) Use of information and communication technologies. In each session, field notes were taken anonymously, and no recordings were made. The intervention consisted of a 10 to 50-minutes face-to-face session at the hospital or at the participants' home, following the principles of collaborative and evocative motivational interview [27–30], favoring the participant's autonomy. The techniques used were open questions, active listening, empathy, returning reflected thoughts, exploring change in goals, summarizing and giving feedback. Also during the enrollment visit, patients were given verbal and written explanation on how to use the app". "During follow-up, the MyPathway® app was used by study participants for bi-directional interaction with the research team. It consisted of positive feedback or reinforcement messages in response to the number of hours of NIV use filled by the patient in a daily basis. In addition, general advice on specific NIV clinical problems was automatically provided by the app according to patients' weekly input. Additional educational material on physical activity, diet and sleep hygiene could be accessed at any time from a dedicated link". See also Multimedia appendix 1 for more technical details		
**5-ix) Describe use parameters**		
**5-x) Clarify the level of human involvement**		
**5-xi) Report any prompts/reminders used**		
"the MyPathway® app was used by study participants for bi-directional interaction with the research team. It consisted of positive feedback or reinforcement messages in response to the number of hours of NIV use filled by the patient in a daily basis" after getting an SMS remainder		
**5-xii) Describe any co-interventions (incl. training/support)**		
"The behavioral mHealth intervention assessed in the current manuscript included: i) a face-to-face motivational interview by a psychologist (EA) to assess patient's adherence profile and lifestyle habits;"		
**6a) CONSORT: Completely defined pre-specified primary and secondary outcome measures, including how and when they were assessed**		
"The primary outcome was a change in self-efficacy measured by the Self Efficacy in Sleep apnea (SEMSA) questionnaire. The SEMSA is a US-designed self-report questionnaire comprising 26 items rated from 1 to 4 on a 4-point Likert scale. The arithmetic mean of the Likert rating for each participant is computed for the overall SEMSA score and for each of the three factors. The total score ranges from 1 to 4. Higher scores indicate greater risk perception, higher benefit expectancy with treatment and greater perceived self-efficacy.<br>Secondary outcomes included: i) usability of the ICT tool measured by the System Usability Scale; ii) patient satisfaction measured using the Net Promoter Score alongside three custom made general satisfaction questions using a Likert scale; iii) continuity of care using the Nijmegen continuity of care questionnaire; and, iv) the Person centered coordinated experience questionnaire as described by Leijten et al. Moreover, ventilator-specific data such as mean hours of use per day, unintentional leaks (L/s), minute ventilation (L/min), tidal volume (mL) and back-up rate (breaths/min) were directly downloaded from the NIV machine.<br>Tertiary outcomes included mortality, health-related quality of life (using the EuroQol 5D questionnaire and sleepiness (using the Epworth Sleepiness Score).<br>The impact of the motivational mHealth tool recommendations on diet and exercise was indirectly measured by body weight changes.<br><br>All assessments were done at baseline and at three months in the final visit programmed in the outpatient clinic"		
**6a-i) Online questionnaires: describe if they were validated for online use and apply CHERRIES items to describe how the questionnaires were designed/deployed**		
**6a-ii) Describe whether and how "use" (including intensity of use/dosage) was defined/measured/monitored**		
**6a-iii) Describe whether, how, and when qualitative feedback from participants was obtained**		
**6b) CONSORT: Any changes to trial outcomes after the trial commenced, with reasons**		
"At time of enrollment, semi-structured motivational interviews were conducted individually. Participants were asked about the following topics: (i) Treatment adaptation experience, (ii) Lifestyle (physical activity and food habits) and (iii) Use of information and communication technologies. In each session, field notes were taken anonymously, and no recordings were made. The intervention consisted of a 10 to 50-minutes face-to-face session at the hospital or at the participants' home". "During follow-up, the MyPathway® app was used by study participants for bi-directional interaction with the research team. It consisted of positive feedback or reinforcement messages in response to the number of hours of NIV use filled by the patient in a daily basis.". "A web-based clinical portal enabled the research team to monitor the NIV hours of use and clinical problems reported by the patients". "a dedicated nurse (one of the authors, MM) took the role of case manager, with clinical and technical knowledge, in order to support collaborative work. She used the web-based portal to identify adherence problems and, accordingly, she contacted the participants via telephone or at home (for those with severe mobility problems) in order to enquire about potential problems, either clinical or technical and solve them."		
**7a) CONSORT: How sample size was determined**		
**7a-i) Describe whether and how expected attrition was taken into account when calculating the sample size**		
**7b) CONSORT: When applicable, explanation of any interim analyses and stopping guidelines**		
"The primary outcome was a change in self-efficacy measured by the Self Efficacy in Sleep apnea (SEMSA) questionnaire. The SEMSA is a US-designed self-report questionnaire comprising 26 items rated from 1 to 4 on a 4-point Likert scale. The arithmetic mean of the Likert rating for each participant is computed for the overall SEMSA score and for each of the three factors. The total score ranges from 1 to 4. Higher scores indicate greater risk perception, higher benefit expectancy with treatment and greater perceived self-efficacy.<br>Secondary outcomes included: i) usability of the ICT tool measured by the System Usability Scale; ii) patient satisfaction measured using the Net Promoter Score alongside three custom made general satisfaction questions using a Likert scale; iii) continuity of care using the Nijmegen continuity of care questionnaire; and, iv) the Person centered coordinated experience questionnaire as described by Leijten et al. Moreover, ventilator-specific data such as mean hours of use per day, unintentional leaks (L/s), minute ventilation (L/min), tidal volume (mL) and back-up rate (breaths/min) were directly downloaded from the NIV machine.<br>Tertiary outcomes included mortality, health-related quality of life (using the EuroQol 5D questionnaire and sleepiness (using the Epworth Sleepiness Score).<br>The impact of the motivational mHealth tool recommendations on diet and exercise was indirectly measured by body weight changes.<br><br>All assessments were done at baseline and at three months in the final visit programmed in the outpatient clinic"		
**8a) CONSORT: Method used to generate the random allocation sequence**		
"The randomization scheme was generated by using the website Randomization.com by one of the researchers (EB), prior to patient enrollment. Blocks of 4 were used. Only after the participant signed the informed consent, the investigator opened the envelope with the allocated study group."		
**8b) CONSORT: Type of randomisation; details of any restriction (such as blocking and block size)**		
"The randomization scheme was generated by using the website Randomization.com by one of the researchers (EB), prior to patient enrollment. Blocks of 4 were used. Only after the participant signed the informed consent, the investigator opened the envelope with the allocated study group."		
**9) CONSORT: Mechanism used to implement the random allocation sequence (such as sequentially numbered containers), describing any steps taken to conceal the sequence until interventions were assigned**		
"The randomization scheme was generated by using the website Randomization.com by one of the researchers (EB), prior to patient enrollment. Blocks of 4 were used. Only after the participant signed the informed consent, the investigator opened the envelope with the allocated study group."		
**10) CONSORT: Who generated the random allocation sequence, who enrolled participants, and who assigned participants to interventions**		
"The randomization scheme was generated by using the website Randomization.com by one of the researchers (EB), prior to patient enrollment. Blocks of 4 were used. Only after the participant signed the informed consent, the investigator opened the envelope with the allocated study group."		
**11a) CONSORT: Blinding - If done, who was blinded after assignment to interventions (for example, participants, care providers, those assessing outcomes) and how**		
**11a-i) Specify who was blinded, and who wasn't**		
"Due to the nature of the intervention, neither the participants nor the investigators in direct contact with them were blinded. Only the investigator in charge of data analysis was blinded."		
**11a-ii) Discuss e.g., whether participants knew which intervention was the "intervention of interest" and which one was the "comparator"**		
**11b) CONSORT: If relevant, description of the similarity of interventions**		
Not relevant		
**12a) CONSORT: Statistical methods used to compare groups for primary and secondary outcomes**		

"Accepting an alpha risk of 0.05 and a beta risk of 0.2 in a two-sided test, 31 subjects were necessary in the intervention group and 31 in the control group to recognize as statistically significant a difference greater than or equal to 0.35 units in the SEMSA overall score. The common standard deviation was assumed to be 0.46 [37]. It was anticipated a drop-out rate of 10%.<br>Baseline and end-of-study data (questionnaires) were collected face-to-face at the outpatient clinic by the investigators (EB, EA and MM). Study data were collected and managed using the REDCap electronic case report form [38, 39] hosted at Hospital Clínic of Barcelona. Data on NIV use and clinical problems with NIV was collected on-line during the study period as reported by the participants using MyPathway®.<br>Results are presented as mean (SD) or n (%) when indicated. Comparisons were done using chi-square or Fisher exact tests for categorical variables, and Student' or Wilcoxon tests, depending on the distribution of the variables, for numerical variables."		
**12a-i) Imputation techniques to deal with attrition / missing values**		
Attrition rate expected was very low since all were long-term patients in the clinical service, known well by the physcians participating in the study. This was reflected by the fact that only 1 patient in the intervention group discontinued due to deteriorating health condition not related to the intervention or respiratory pathology but from a surgical complication.		
**12b) CONSORT: Methods for additional analyses, such as subgroup analyses and adjusted analyses**		
We did not do any additional analysis		
RESULTS		
**13a) CONSORT: For each group, the numbers of participants who were randomly assigned, received intended treatment, and were analysed for the primary outcome**		
As seen in the CONSORT flow diagram all patients in the intervention group (except for one drop-out) downloaded the app and used it. All received face-to-face motivational interview at enrolment. All were actively followed by the dedicated nurse using the web portal, and if deemed necessary, phone contact and/or home visit.		
**13b) CONSORT: For each group, losses and exclusions after randomisation, together with reasons**		
In Multimedia Appendix 3 - CONSORT flow diagram the exclusion and reasons are stated. In the control group there were no loses. In the intervention group only one lost due to deteriorating health condition.		
**13b-i) Attrition diagram**		
**14a) CONSORT: Dates defining the periods of recruitment and follow-up**		
"Between February and March 2019"		
**14a-i) Indicate if critical "secular events" fell into the study period**		
**14b) CONSORT: Why the trial ended or was stopped (early)**		
The trial was ended or stopped early		
**15) CONSORT: A table showing baseline demographic and clinical characteristics for each group**		
"Baseline demographic and clinical characteristics are shown in Table 1 and Multimedia Appendix 2."		
**15-i) Report demographics associated with digital divide issues**		
In table 1 and multimedia appendix 2, the following demographics are reported: sex, gender, educational level		
**16a) CONSORT: For each group, number of participants (denominator) included in each analysis and whether the analysis was by original assigned groups**		
**16-i) Report multiple "denominators" and provide definitions**		
In multimedia appendix 2, "NIV usage reported by patients using MyPathway" details the use of the app for reporting and "MyPathway logobook analysis" report the absolute and relative numbers for different aspects of the usage.		
**16-ii) Primary analysis should be intent-to-treat**		
**17a) CONSORT: For each primary and secondary outcome, results for each group, and the estimated effect size and its precision (such as 95% confidence interval)**		
"For the primary outcome, there was no statistically significant difference after intervention in the SEMSA score for self-efficacy (mean[SD]=3.4[0.6] vs 3.4 [0.5], P=.51).<br>For the perceived risks, outcome expectancies, Epworth Sleepiness Score and EuroQol 5Q-5D questionnaires, there were no statistically significant differences neither (see Multimedia Appendix 2). As for the patient experience questionnaires, neither the Nijmegen continuity of care questionnaire nor the Person centred coordinated experience questionnaire showed statistically significant differences between groups (see Multimedia Appendix 2)". "Adherence (i.e. number of hours of use/d of NIV as recorded by the ventilators) showed no difference after intervention (mean[SD]=7.4[2] vs 7.7[2]). The only ventilatory parameter showing a statistically significant difference after three months in the intervention group was the minute ventilation (mean[SD]=7.0[2] vs 6.4[2.1], P=.03). The remaining ventilatory parameters and weight are shown in the Multimedia Appendix 2. None of the patients died during the trial."		
**17a-i) Presentation of process outcomes such as metrics of use and intensity of use**		
**17b) CONSORT: For binary outcomes, presentation of both absolute and relative effect sizes is recommended**		
We did not have binary outcomes		
**18) CONSORT: Results of any other analyses performed, including subgroup analyses and adjusted analyses, distinguishing pre-specified from exploratory**		
We did not conduct subgroup or adjusted analysis		
**18-i) Subgroup analysis of comparing only users**		
**19) CONSORT: All important harms or unintended effects in each group**		
"None of the patients died during the trial."		
**19-i) Include privacy breaches, technical problems**		
**19-ii) Include qualitative feedback from participants or observations from staff/researchers**		
DISCUSSION		
**20) CONSORT: Trial limitations, addressing sources of potential bias, imprecision, multiplicity of analyses**		
**20-i) Typical limitations in ehealth trials**		
"We do acknowledge that by using an already existing app, the co-design phase was skipped. Also we did not measure technological literacy in our older population (average age 69 years)". "a clear limitation of our study was the exclusion of new NIV patients, were the behavioral intervention may have had more impact"		
**21) CONSORT: Generalisability (external validity, applicability) of the trial findings**		
**21-i) Generalizability to other populations**		
"Our study considered the whole population of patients attending the clinic, making it a realistic clinical scenario"		
**21-ii) Discuss if there were elements in the RCT that would be different in a routine application setting**		
**22) CONSORT: Interpretation consistent with results, balancing benefits and harms, and considering other relevant evidence**		
**22-i) Restate study questions and summarize the answers suggested by the data, starting with primary outcomes and process outcomes (use)**		
"We report the results of a behavioral mHealth intervention based on a face-to-face interview and the use of a mHealth tool (MyPathway® app), during a follow-up period of three months, on patients with hypercapnic chronic respiratory failure under home-based long-term noninvasive ventilation. To the best of our knowledge, this is the first randomized controlled trial using digital tools to support behavioral changes in this population"."In the current study, the self-efficacy mean score was already high at baseline (Table 1) and we did not find any significant effect on behavioral changes after the intervention". "Notwithstanding the clinical results, it is important to note that the mHealth tool was well appreciated by the patients and their family/caregivers. "."In this respect, we measured two process outcomes [50] related to patient experience [33], i.e. continuity of care and person-centered care. Both parameters were very well evaluated by all of our study population, which included not only patients, but also their family and carers in a third of cases in the intervention group."		
**22-ii) Highlight unanswered new questions, suggest future research**		
Other information		
**23) CONSORT: Registration number and name of trial registry**		
NCT03932175 (clinicaltrials.gov, April 30, 2019)		
**24) CONSORT: Where the full trial protocol can be accessed, if available**		
Full protocol was not published previously		
**25) CONSORT: Sources of funding and other support (such as supply of drugs), role of funders**		
This work was supported by the European Union's Horizon 2020 Research and Innovation Programme under grant agreement n° GA-689802 (CONNECARE).		
**X26-i) Comment on ethics committee approval**		

**x26-ii) Outline informed consent procedures**		
**X26-iii) Safety and security procedures**		
**X27-i) State the relation of the study team towards the system being evaluated**		
